# The use of nanocrystal quantum dot as fluorophore reporters in molecular beacon-based assays

**DOI:** 10.1186/s40580-016-0094-6

**Published:** 2016-12-01

**Authors:** Oluwasesan Adegoke, Enoch Y. Park

**Affiliations:** 1 Laboratory of Biotechnology, Research Institute of Green Science and Technology, Shizuoka University, 836 Ohya, Suruga-ku, Shizuoka, 422-8529 Japan; 2 Laboratory of Biotechnology, Department of Bioscience, Graduate School of Science and Technology, Shizuoka University, 836 Ohya, Suruga-ku, Shizuoka, 422-8529 Japan

**Keywords:** Molecular beacon, Quantum dots, Nanocrystal, Biosensor

## Abstract

The utilization of molecular beacon (MB) biosensor probes to detect nucleic acid targets has received enormous interest within the scientific community. This interest has been stimulated by the operational qualities of MB-based probes with respect to their unique sensitivity and specificity. The design of MB biosensors entails not only optimizing the sequence of the loop to hybridize with the nucleic acid target or optimization of the length of the stem to tune the sensitivity but also the selection of the appropriate fluorophore reporter to generate the signal transduction read-out upon hybridization of the probe with the target sequence. Traditional organic fluorescent dyes are mostly used for signal reporting in MB assays but their optical properties in comparison to semiconductor fluorescent quantum dot (Qdot) nanocrystals are at a disadvantage. This review highlights the progress made in exploiting Qdot as fluorophore reporters in MB-based assays with the aim of instigating further development in the field of Qdot-MB technology. The development reported to date indicates that unparalleled fluorescence signal reporting in MB-based assays can be achieved using well-constructed Qdot fluorophores.

## Background

Our post-genomic age is experiencing a rapid surge in advanced quantitative research involving the development of diagnostic probes to derive genomic information, diagnose and prevent the rapid spread of disease. The quality of research output with the use of advanced biomolecular probes in generating unprecedented sensitivity and specificity for nucleic acid detection has inspired several researchers to explore ways of designing sophisticated and smart biosensor probes. A classical example of such biosensor probe for quantitative genomic analysis and nucleic acid detection is molecular beacon (MB) [1–8]. MB technology was first discovered in 1996 by Tyagi and Kramer [7]. They are DNA oligonucleotide probes specifically designed in a stem-loop hairpin structure and embedded with a quencher-fluorophore reporter pair. The loop of the MB is designed to hybridize a complimentary DNA base pair to the target nucleic acid. In the absence of the target nucleic acid, the MB is constructed in a hairpin structure, thereby forcing the quencher into close proximity with the fluorophore molecule. Under this conformation, the fluorescence of the MB is “switched OFF” while upon hybridization of the loop sequence with a complimentary nucleic acid target, the hairpin stem-loop structure opens up, thus separating the quencher from the fluorophore and the fluorescence is “switched ON”.

The transition between a MB fluorescence “switch OFF” and “switch ON” state enables the differentiation between unbound and bound probes with background signal-to-noise ratio that could exceed 200, hence making it an ultrasensitive probe for nucleic acid detection [9–15]. With respect to specificity, MB stands out amongst other linear oligonucleotide probes [2]. The hairpin, which can be considered a secondary structure of an oligonucleotide has long been recognized to generate improved specificity [16]. In the presence of unspecific polymerase chain reaction (PCR) byproducts, the unique specificity of MB allows for the desired amplicon to be recognized. This unique property enables MB to the highly desirable in clinical assays for disease diagnosis and detection.

Other potential applications of MB are the quantification and detection of genomic disease markers in tissues and living cells. Conventional technologies such as real-time PCR, Northern blotting, expressed sequence tags, DNA microarrays and serial analysis of gene expression are not capable of generating temporal data about gene expression in biological systems [16]. Whereas, the MB can be used to study the processes of gene expression in biological systems in response to external stimuli such as hormones, drug molecules and growth factor, thus generating a wealth of useful information on disease and health.

A key component to be considered in the structure of a MB is the fluorophore reporter. The function of the fluorophore reporter is to generate the output signal depending on the molecular changes within the MB structure. The hybridization of the loop oligonucleotide sequence with the target nucleic acid induces the fluorophore reporter to transduce an optical signal which is directly proportional to the concentration of the target. The choice of fluorophore reporter is crucial in influencing the sensitivity of the MB. Highly sensitive fluorophore reporters are needed to generate unprecedented sensitivity when utilizing MB as diagnostic probes for nucleic acid detection and diagnosis. Most of the fluorophore reporters used in MB assays are fluorescent organic dyes but there are limitations with respect to their optical properties.

Fluorescent inorganic semiconductor quantum dot (Qdot) nanocrystals are example of fluorophore reporters that have been used in MB design. The unique optical properties of Qdots have made them distinguishable from other fluorophores such as organic fluorescent dyes. Little attention has been paid to the exploitation of Qdot as fluorophore reporters in MB assay. It is important to emphasis that the selection of a fluorophore reporter is crucial in generating robust sensitivity in MB assay. In this review, we discuss the basic design factors of MB, a discussion on the optical properties of Qdots and the advantages they hold over fluorescent organic dyes. The applications of Qdot-MB biosensors in diagnosis and detection were reviewed and the future outlook for Qdot-MB based biosensors is suggested. Generally, it is impossible to review all MB-related detection and diagnosis in this paper as we have limited our attention to Qdot-MB based biosensors. Several reviews have outlined MB technology for different type of applications which the readers of this paper can refer to [17–20]. Our motive here is to draw attention to the progress made in the design of Qdot-based MB biosensors as we aim to encourage further development in this field and to generate broader interest in Qdot-MB technology.

## Factors influencing the design of MBs

**Fig. 1:**
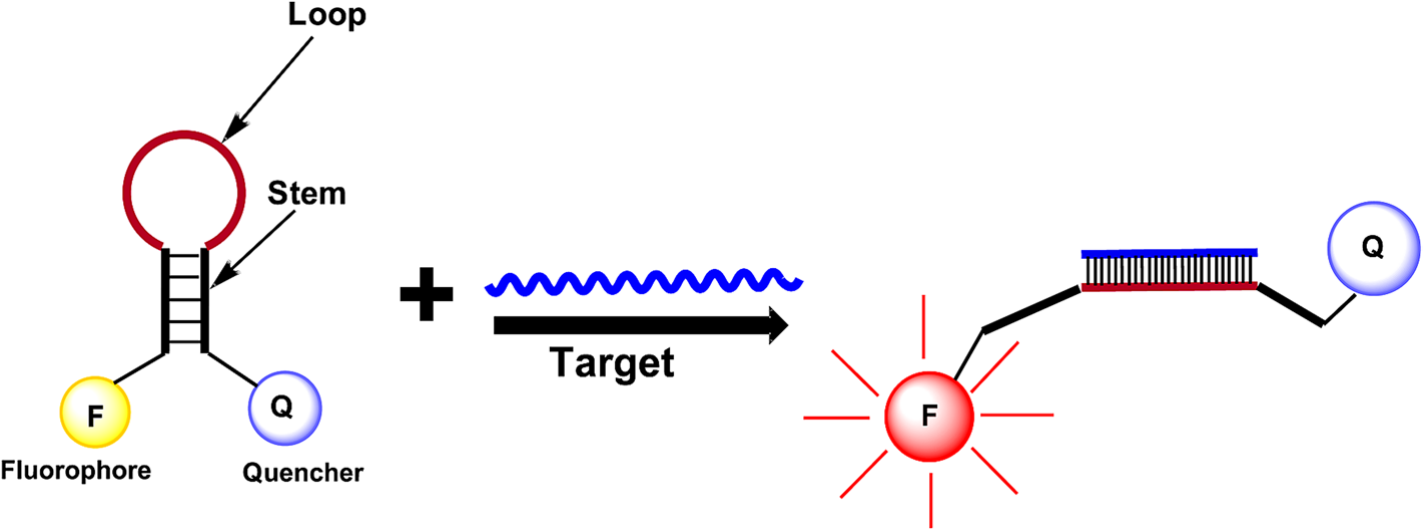
A classic example of the structure of a MB and the corresponding working principle

The design of a MB involves four essential components: stem, loop, quencher and fluorophore, as shown in Fig. 1. The stem is formed from two complimentary short arm base pairs which are independent of the target nucleic acid sequence while its length could be four to six complimentary sequences. Flexibility in MB design can also enable the arm of the stem to participate in both target hybridization and stem formation. The loop is typically made up of 15–25 base pairs and it is discreetly selected based on the sequence being targeted. Despite the possibility of being able to label a MB with any selected quencher-reporter pair, appropriate selection of the quencher and fluorophore reporter could allow for multiplexing capabilities and also aid the improvement of background signal-to-noise ratio. The most vital design factors of MBs are the stem length, nucleotide sequence and probe. At any given temperature, these factors are responsible for controlling the different conformational states of the MB with respect to random coil, stem-loop and bound-to-target [21]. Target-specific probe design is a prerequisite for many applications, i.e. a single nucleotide polymorphism being surrounded by the probe sequence. Optimization of the performance of a MB with respect to background-signal-to-noise ratio, hybridization rate and specificity for a target application is dependent on the stem sequence, stem length and the probe (fluorophore-quencher pair).

It has been demonstrated that discrimination of mutant target and wild-type across a broad range of temperatures is influenced by longer stem length of the MB. This may be induced by the improved stability (lower free energy) of the MB stem-loop structure. The reduced free energy between MB target duplexes and unbound MB, creates an environment where the energetic choice of target-specific binding is reduced by a single base mismatch [16]. This may directly explain why linear probe exhibits lower specificity in comparison to MB probes. Decreased MB-target hybridization rate and lower target affinity are features of the use of longer stem lengths. Conversely, improved target affinities and faster hybridization rates are features of MB with shorter stem lengths. However, MBs with longer stem length exhibit higher background signal-to-noise ratio than MBs with shorter stem [16]. It is important to emphasize that MB without a stem and lacking the complementary arm base pairs depends solely on hydrophobic interaction and random-coiled nature between the quencher and fluorophore molecule to maintain the fluorescence “switch OFF” state and to distinguish between unbound state and bound state. The effects of stem length on the efficiency of MB are more important than the probe length and this can be used to tweak the functionality of the MB.

The choice of fluorophore reporter for a MB is crucial in obtaining ultrasensitive detection of the target nucleic acid, multiplex detection characteristics and generating high background signal-to-noise ratio. Most MBs utilize conventional organic fluorescent dyes as fluorophore reporter. Although, the sensitivity of a MB is not only reliant on the reporter molecule but also on the binding efficiency of the probe. A highly sensitive fluorophore reporter and the strong binding efficiency of the probe equate to an ultrasensitive MB biosensor for the target nucleic acid. With respect to binding efficiency, this is dependent on the affinity of the probe to the target nucleic acid. For the choice of fluorophore reporter, most commonly used organic dye molecules are; fluorescein (Fam), tetrachloro-6-carboxyfluorescein (Tet), 5-(2′-aminoethyl) aminonapthalene-1-sulfonic acid (EDNAS), hexachloro- 6-carboxy fluorescein (Hex), tetramethylrhodamine (Tamra) and 5-carboxyrhodamine-X (Rox). Semiconductor fluorescent Qdots have also been utilized as fluorophore reporter in MB design but to a lesser degree than organic fluorophore dyes. Although, the optical properties of Qdots are far superior to organic fluorescent dyes [22–25], it is quite surprising that less attention has been given to the use of Qdots in MB design. This review focuses on highlighting the progress made in the use of Qdot in MB biosensors and to pinpoint how the optical properties of Qdots can be engineered to produce high quality nanocrystals that can yield unparalleled signal in MB applications.

Another factor to consider in MB design is the choice of quencher molecule. The quencher can produce higher background signal-to-noise ratio, although to a lesser extent than the fluorophore reporter. Quenchers play a crucial role in modulating the detection mechanism of the MB. Most notable quenchers used are organic dark quenchers such as blackhole quencher (BHQ), Iowa black and 4-((4-(dimethylamino)phenyl)azo)benzoic acid (DABCYL) [16]. These quenchers can effectively quench a wide range of fluorophore molecules. The quenching mechanism of the fluorescence of the fluorophore molecule by an adjacent quencher is based on fluorescence resonance energy transfer (FRET) or static quenching (also known as contact quenching). For FRET process, the quencher (acceptor) must be fluorescent, in which the energy from the fluorophore molecule (donor) is transferred to the quencher. The energy transfer rate is influenced by the distance between the donor and quencher molecule and the effect is reflected by the quenching of the fluorophore molecule and apparent fluorescence enhancement in the quencher molecule (due to energy transfer). However, this basic theory is not always the case, as a number of papers have reported the quenching of the fluorescence of fluorophore molecules by non-fluorescent quenchers [26] and have attributed the quenching process to FRET. Most quenchers used in MB design are non-fluorescent dark quenchers. These quenchers do not exhibit fluorescence properties and our group have recently reported that they quench the fluorescence of Qdot-MB probes by static quenching [27]. In addition to the aforementioned quencher molecules, gold nanoparticles (AuNP) have also been used as a quencher molecule in MB design [28].

### Factors influencing MB operation

There are several factors that influences the operational performance of MB-based probes. The distance between the quencher-fluorophore pair is the primary factor that determines the quenching efficiency of the probe [21]. Based on the concept of FRET, the energy transfer efficiency is proportional to the 6th power of the distance between the donor–acceptor pair. Studies have shown that efficient energy transfer can yield 10–100-fold increase in fluorescence intensity upon hybridization [2].

Another factor which influences MB operation is temperature. At low temperature, a stable hairpin structure of the MB can be maintained but distortion of the structure can occur at higher temperature [29]. The chain length of the stem influences the melting temperature, ionic strength of the buffer and on the guanine-cytosine content. Bonnet et al. reported the effect of temperature on a MB with perfect complementary sequence [21]. At low temperature, a complementary duplex hybrid between the loop sequence of the MB and the target sequence was formed, thus generating strong fluorescence recovery signal. As the temperature was gradually increased, the duplex hybrid formed between the MB and the target sequence was broken and the MB returned to its original hairpin state resulting in a weakened fluorescence signal. With further increase in the temperature, the hairpin structure of the MB was completely destroyed, resulting in a linear structure and restoration of the fluorescence.

The pH of the MB probe solution is also a factor that influences its performance. At high pH, the stem of the MB is broken and this degrades the MB leading to a false negative signal [2].

### MB synthesis

The synthesis MB mimics the synthesis of dual-labeled oligomers with two dyes [30]. The loop sequence length (15–40 bp nucleotide) is selected so as to ensure a stable target-probe hybrid at the chosen temperature. The choice of the stem length (5–7 bp nucleotide) should be appropriated such as it forms a stable hairpin stem-loop structure for effective fluorescence quenching and should also be weak enough to dissociate when a duplex hybrid is formed between the probe and the target sequence.

The most commonly used quencher is DABCYL, known as a universal quencher due to its ability to quench a wide range of fluorescent-emitting fluorophores [2]. Hence, it is usually used as a starting material for MB synthesis via a “DABCYL-controlled pore glass (CPG)” [30]. A variety of fluorescent dye molecules of choice can be directly linked to the 5′ end of the probe to report the fluorescence signal based on the structural change in probe structure.

There are four main steps involved in the synthesis of MBs [30]; Firstly, DABCYL is derivatized with the CPG solid support and used to initiate the synthesis at the 3′ end. Afterward, sequential addition of the rest of the nucleotide is performed using standard cyanoethylphosphoramidite technique. Secondly, at the 5′end, a primary amine group is linked. Thirdly, hydrolysis of the oligonucleotide is performed followed by removal from the CPG and finally purified by reverse-phase liquid chromatography. Lastly, labelling of the purified oligonucleotide with a fluorophore can be performed and the excess fluorophore dye is then removed by gel chromatography on a Sephadex G-25 column or a Qdot fluorophore can be directly conjugated to the MB [27].

### Advantages of MB over other nucleic acid probes

Several DNA fluorescence probes have employed the hybridization technology as a means to target a complementary sequence. Nucleic acid blotting assay is a classic example of techniques employing hybridization technology. This technique has contributed to the understanding of gene technology. The technique operates by using a solid support to incorporate DNA fragments and an oligonucleotide probe embedded with the target sequence is used to aid hybridization [30, 31]. However, for real-time monitoring of nucleic acid synthesis and nucleic acid labeling in living cells, nucleic acid blotting assays, or probes using target-probe isolation or other assays using intercalating reagents cannot be used for such applications.

Fluorescence transduction signal mechanism, which is influenced by changes within the chemical environment of a fluorophore molecule, has enabled MB to function as ultrasensitive probes for real-time monitoring whilst generating high background signal-to-noise ratio. The fluorescence “switch ON” state of a MB can increase up to 200-fold when hybridized with the target nucleic acid [2]. Hence, with respect to signal generation, MB holds the advantage over other oligonucleotide probes. This is corroborated by the imaging of single molecule DNA in living system [32]. MB possesses the functionality of being effective in situation where it is practically difficult or undesirable to separate the target-probe hybrid from an excess of unhybridized solution. This is essentially needed in the detection of mRNAs in living cells or in real-time PCR in sealed tubes [30]. We cannot overemphasize the degree of usefulness of MB in detection without the separation of the target-probe. This process enables the direct monitoring of nucleic acids during synthesis in living samples or in sealed tubes, without the need for further manipulation.

The molecular specificity of MB is another unique advantage. MBs are extremely target specific and are practically effective in single nucleotide polymorphism (SNP) detection. Conventional technique for SNP detection is time consuming and labor-intensive but MBs offer a simple and direct means for genetic disease diagnosis and in gene therapy studies [5]. Studies have shown that hybridization from perfect complimentary DNA sequence and nonhybridization from mismatch DNA sequence occur at a wider temperature for MB probes than for traditional oligonucleotide linear probes [21].

## Qdots

Fluorescent semiconductor Qdots have attracted immense research attention within the last three decades. Qdots are 0-dimensional nanocrystals in comparison to bulk materials and their minimal number of electrons translate to quantized discrete energy like-state giving rise to a nonagglomerated 0-dimensional nanostructure [33, 34]. Even though Qdots are considered to be 0-dimensional, they are referred to as a box in quantum mechanics in which the optical properties are unique to each size [35]. One of the most unique properties of Qdots is the quantum confinement effect which is reflected by the blue shifting of the energy band gap when the nanocrystal diameter increases relative to the nature of the semiconductor metals that composes its structure. Another reflection of quantum size effect is the emergence of discrete atomic separated energy state due to the presence of smaller atoms [35].

The most commonly used class of fluorescent Qdots in sensing and biosensing applications are the group II-VI semiconductor chalcogenide materials. These includes the cation of cadmium, mercury and/or zinc combined with anionic metals, like sulfur, tellurium, and selenium. Amongst the available Qdot nanocrystals, CdS, CdTe, CdTe/ZnS, CdSe and CdSe/ZnS-based are the most studied and utilized as fluorophore reporters in sensor/biosensor design. They can be classified as conventional Qdots and they are also commercially available. Cd-based Qdots have mostly been exploited as fluorophore reporters due their unique optical properties which are far superior to organic fluorescent dyes. For conventional Qdot systems, their optical properties can be tuned directly by size or monolayer of shell growth.

Core CdTe and CdSe-based Qdots suffer from low fluorescence quantum yield (QY) and poor fluorescence stability due to surface defect arising from dangling bonds which act as trap sites on their surface. A general and well adopted means of improving the optical properties of the Qdots is passivation of its surface with a shell material. Several shell materials ranging from binary to alloyed ternary semiconductor metal components have been overcoated on the surface of the core Qdots [36–43]. In MB biosensors, the most widely used Qdot as fluorophore reporter is CdSe/ZnS due to its popularity and well reported improved optical properties, as a result of the ZnS passivating effect. Despite the popularity of CdSe/ZnS Qdots, the large lattice mismatch between the core and shell materials can introduce interfacial strain and dislocation which ultimately lowers its PL QY. Despite this anomaly, CdSe/ZnS Qdots still possess exceptional optical properties than organic fluorescent dyes.

**Fig. 2:**
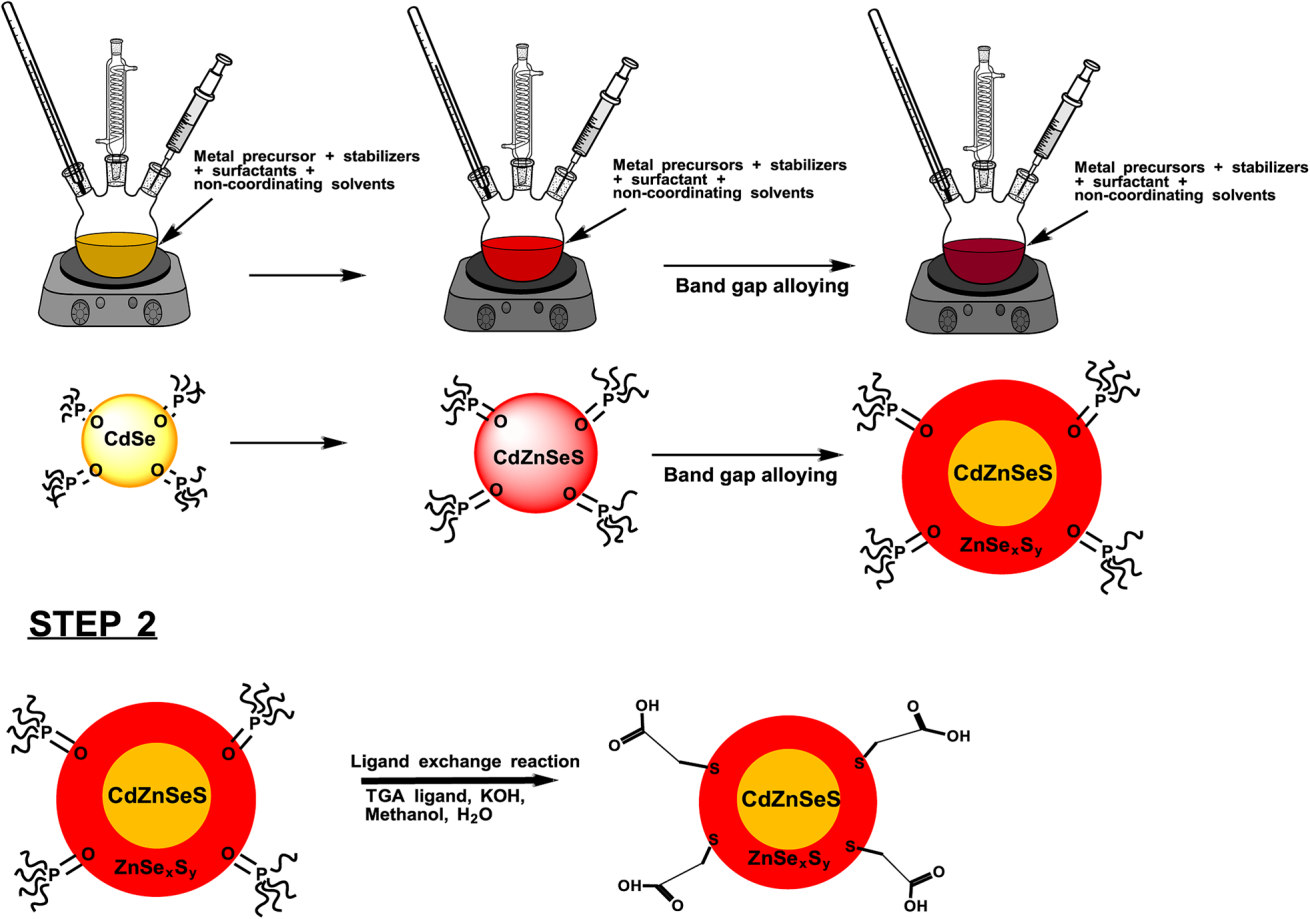
Synthetic scheme showing the bandgap engineered alloying of CdZnSeS/ZnSe_x_S_y_ Qdots and the subsequent ligand exchange reaction with TGA [46]

The ability to fine-tune the structure of Qdots makes it possible to develop new generation Qdot systems with unique optical properties. Particularly, alloying of the Qdot structure is another means to engineer their optical properties. This involves varying the stoichiometric metal composition to tune the optical properties of the Qdots. Qdots with unprecedented luminescent properties can be developed by alloying processes [44, 45]. Figure 2 shows a descriptive synthesis scheme for the band gap engineering of alloyed CdZnSeS/ZnSe_x_S_y_ Qdots by which the stoichiometric molar fraction of Se to S was varied. Step 1 shows the synthetic fabrication step for the hydrophobic CdZnSeS/ZnSe_x_S_y_ Qdots while step 2 shows the ligand exchange reaction to replace the organic capping with thioglycolic (TGA) ligand. The advantage of such fabrication technique provides the means to tune to optical properties of the Qdots without changing the size and also ensuring that high PL QY can be obtained. In this review, we highlight the strides made using both conventional and new-generation Qdots as fluorophore reporters in MB assays.

### Comparison of the optical properties of Qdots and fluorescent organic dyes

Organic fluorescent dyes can be classified as traditional fluorophores used in MB biosensor design. Subsequently, fluorescent Qdots came into the fray and have attracted interest due to their unique optical properties and biocompatibility. Organic fluorescent dyes are generally bonded to the MB during synthesis whilst Qdots nanocrystals are directly conjugated to the MB in solution. In this section, we compare the optical properties of semiconductor Qdot nanocrystals with those of organic fluorescent dyes.

It is important to emphasize that the optical properties of Qdots arise from interactions between hole and electron within their local chemical environments. The chemistry associated with the fluorescence of Qdots involves the excitation of electron from the valence band to the conduction band, hence creating a hole in the valence band. With respect to the emission line width, those of Qdots are narrower than organic fluorescent dyes. The extent of narrowing or broadening of the emission line width provides information on the quality of size distribution of the materials as well as surface defect states. Hence, narrower emission line width is more favorable and Qdots holds the advantage. Organic fluorescent dyes are highly prone to photobleaching due to degradation of their fluorescence after prolonged exposure to light, whereas Qdots are ~100–200 times more stable and ~20 more brighter [47]. It is estimated that the molar extinction coefficient of Qdots (CdSe), which depends on the excitation wavelength and particle size, range between 10^5^ and 10^6^ M^−1^ cm^−1^ and is 10–100 times larger than those of organic fluorescent dyes [48]. The quantum size effect of Qdots is a unique advantage over organic fluorescent dyes due to the ability to obtain several Qdots size of the same structure and with varying optical properties, thus making them suitable for multiplex detection and the ability to select appropriate Qdot size for the targeted application.

### The chemistry and optimization strategies of Qdots-MB probes

**Fig. 3:**
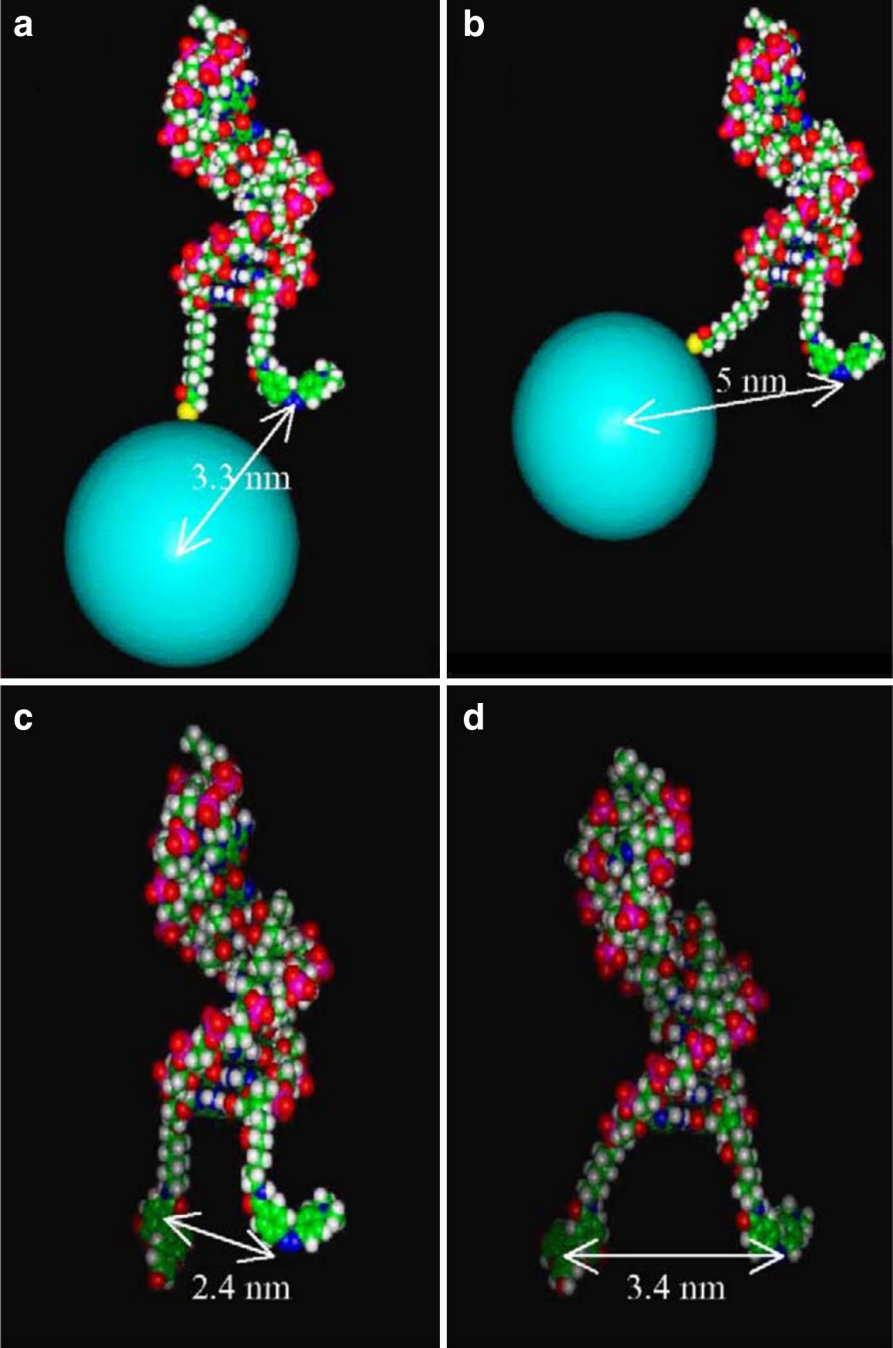
A 3D molecular modeling of Qdot-modified MBs. This model is a snap shot of a highly dynamic macromolecule. Size of Qdot here is 3.7 nm. **a** The nearest possible separation between Qdot and DABCYL, **b** the farthest possible separation between Qdot and DABCYL, **c** the nearest possible separation between the 6-FAM and DABCYL, **d** the farthest separation between the 6-Fam and DABCYL. Reprinted from Ref. [49]. Copyright (2004), with permission from Elsevier

A comparative study was carried out by Kim et al. in [49] in which mercaptoacetic acid (MAA)-capped CdSe/ZnS Qdots was conjugated to the 5′ end of the MB with DABCYL as the fluorescence quencher and alternatively, organic fluorescent dye, 6-FAM was attached to the 5′end, also using DABCYL as the quencher molecule. The interaction between the Qdot or 6-Fam with DABCYL was attributed to FRET, hence the chemical interaction was studied on this basis. A more extended overlap of the fluorescence of the Qdot (90%) with the absorption spectrum of DABCYL was observed over that of 6-Fam (30%). Using molecular modeling simulations, the potential distance between the Qdot/DABCYL and 6-Fam/DABCYL was determined. Figure 3 shows the snap shot of the potential distance (nearest and farthest) between Qdot/DABCYL and 6-Fam/DABCYL (nearest and farthest). It was predicted that the potential distance, nearest and farthest between the Qdot and DABCYL was 3.3 and 5 nm, respectively. The distance was simulated from the N=N bond (center) of DABCYL to the center of the Qdot. The Förster resonance distance was calculated to be 3.4 nm while the FRET efficiency for the farthest and nearest Qdot/DABCYL distance was calculated to be ~9 and ~54%, respectively. The potential nearest and farthest distance between DABCYL and 6-Fam was calculated to be 2.4 and 3.4 nm which was relatively smaller than that for the Qdot/DABCYL distance.

The distance measurement of 6-Fam/DABCYL was measured from the center of 6-Fam (C9 in Xanthene) to the N=N bond of the quencher, DABCYL. The Förster distance was calculated to be 4.6 nm while the FRET efficiency was calculated to be 98%. It was observed that the larger center-to-center spacing between DABCYL and the Qdot was relatively larger than that of 6-Fam/DABCYL. This difference was attributed to the larger size effect of the Qdots which also influenced its FRET efficiency of the Qdots and the Förster radius between the Qdot and DABCYL. Since this study was limited to a single Qdot size, the quantum size effect of Qdots which could yield several sizes across the visible region to the near infra-red will have a profound effect on the interaction with quencher molecules in MB assays.

In another related development, the optimized linkage and quenching effects of different quencher molecule to the MB were studied by Cady et al. when using Qdot as the fluorophore reporter [26]. In their study, different quenchers, DABCYL, Iowa Black and 1.4 nm gold nanoparticles, bonded to the MB was used as a means to study the quenching interaction with the Qdot fluorophore. Carboxyl-modified Qdots were conjugated to the amino-linked 5′end of Iowa Black and DABCYL bonded MBs while streptavidin-functionalized Qdots were bonded to biotinylated MBs with gold nanoparticle and Iowa Black as quencher molecules. It was observed that Iowa Black exhibited a similar quenching efficiency to gold nanoparticle and both were better than DABCYL. With respect to the linkage strategies of the Qdots to the MBs, it was observed that carboxyl-bonded MBs generated better output efficiency than streptavidin-bonded MBs. The difference was attributed to the binding efficiency between the numbers of accessible streptavidin or carboxyl groups on the Qdots. It was suggested that the secondary hairpin structure formation could be impeded by the packing of MB DNA on the Qdots surface and thereafter results in low quenching efficiency of the Qdot (in the absence of the target nucleic acid) and small fluorescence enhancement changes upon hybridization with the target. The relative distance between the quencher molecules and the fluorophore reporter was also suggested to influence the overall performance of the MB probe. In terms of FRET efficiency, carboxyl-functionalized Qdot exhibited an 89% energy transfer rate in comparison to 14% exhibited by streptavidin-functionalized Qdots.

## MB biosensors using Qdots as fluorophore reporter

### DNA detection

The detection of DNA is by far the most common application of Qdot-MB probes. Cady et al. developed a Qdot-MB biosensor for DNA [26] and studied the fluorescence enhancement efficiency based on different linkage strategies and different fluorescence quenchers bonded to the MB. Carboxyl-modified Qdots bonded to Iowa Black or DABCYL-bonded MB and streptavidin-modified Qdots bonded to Iowa Black and gold nanoparticle-bonded MB were used in their study. They observe that the fluorescence increase after hybridization with the target DNA followed the order Carboxyl-Qdot/Iowa Black MB (3.3-fold increase) > streptavidin-Qdot/Iowa Black MB (2.1-fold increase) > streptavidin-Qdot/gold nanoparticle MB (1.9-fold increase) > Carboxyl-Qdot/DABCYL MB (1.0-fold increase).

A novel strategy for MB binding readout for DNA detection involving the separation of the Qdot signal reporter and the molecular recognition element was developed by Wang et al. [50]. A label-free MB-based DNA probe was developed by using an unmodified thymine-rich DNA oligonucleotide probe that is specific to the target DNA and also acting as the captured probe. Hg^2+^ which served as the competitor, binds partially to the capture DNA oligonucleotide signal transducer while the Qdot reporter (MAA-capped CdTe/ZnS) was used as the signal reporter. In the absence of the target DNA, a hairpin structure is formed upon binding of Hg^2+^ to the capture DNA probe. Upon interaction of the target DNA, displacement of the bound Hg^2+^ from the hairpin structure results in binding interaction with the Qdots and the signal read-out is obtained from the Qdot fluorescence. A detection limit of 25 nM for DNA detection was reported. Compared with conventional MB, the label-free MB was proposed to exhibit three notable features; (i) by separating the Qdot signal reporter and the molecular recognition element, the double-labeled formation which could lead to lose of specificity and affinity of the oligonucleotide can be avoided; (ii) release of several Hg^2+^ to interact with the fluorophore reporter can lead to amplified detection signal and (iii) flexibility in the specificity and sensitivity adjustment can be achieved by the Hg^2+^-dependent stem formation. Point (iii) is quite unique because Hg^2+^ was used to modulate the sensitivity and specificity of the MB while for conventional MB probe, optimization of the specificity and sensitivity can be achieved by varying the length of the stem and loop sequence and this will require synthesizing several MBs.

**Fig. 4:**
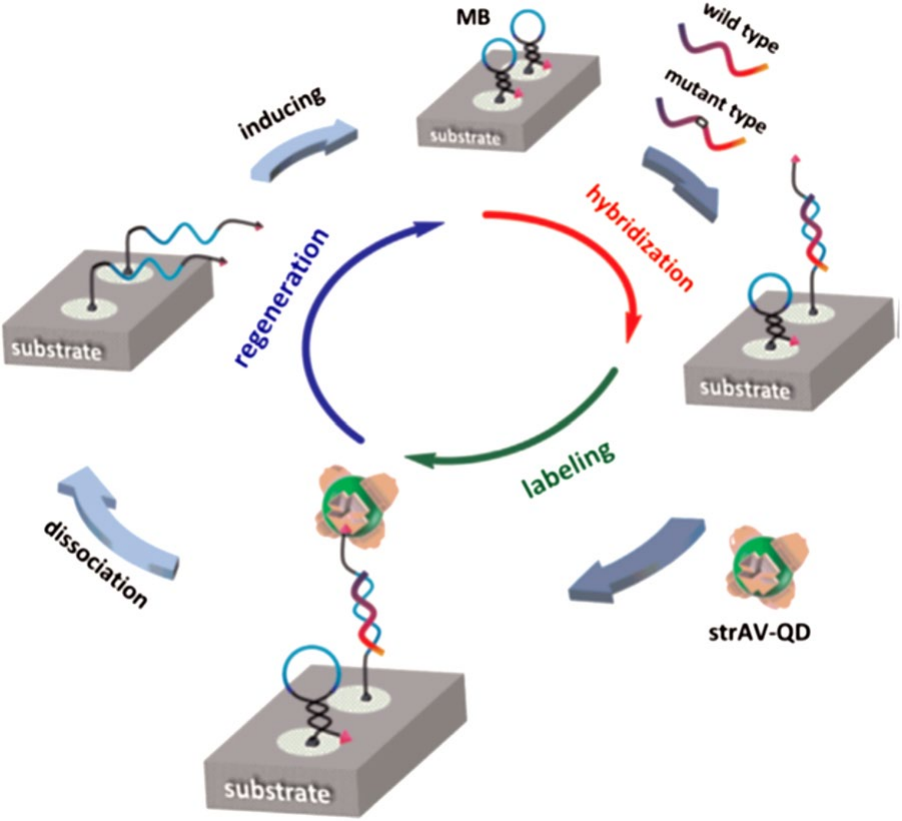
Principle for cyclic detection of target DNA sequence using a MB microarray based on a streptavidin-Qdot (strAV-QD) label. Reprinted from Ref. [51]. Copyright (2016), with permission from Elsevier

A MB microarray assay for label free detection of DNA based on streptavidin-Qdot labeling on the MB instead of the target was developed by Guo et al. [51]. As shown in Fig. 4, their biosensor operates by functionalizing the MB with biotin and a thiol rather than a quencher and a fluorophore at the 3′ end and 5′end of the probe. The microarray was developed by immobilizing the MBs via the epoxy group on the surface of the slide and the 5′end of the terminal thiol group on the MB. In the presence of the target DNA, the hybridization effect triggers the opening of the MB, hence the streptavidin-Qdots label the MB via the biotin-steptavidin interaction. As shown in the Fig. 4, MB1 and MB2 were immobilized on one side of the microarray system. The microarray contained four subarrays in which four genotypes were acquired by mixing two mutant types and two wild types. The single nucleotide polymorphism (SNP) detection was carried out for the four genotypes on the four subarrays. A detection limit as low as 0.1 pM was reported.

A capillary electrophoresis-assisted GSH-CdTe Qdot-MB biosensor was developed for dual single-base DNA mutation detection by Li et al. [52]. To detect the dual single-base mutation, the 15-base pair oligonucleotide loop sequence of the Qdot-MB1 was complementary to the 3′end of the DNA target in which mutation existed at the G position while the 15-base pair loop sequence of the Qdot-MB2 was complementary to the 5′end of the DNA target in which the mutation existed at the T position. The hybridization process was monitored on a capillary electrophoretic device incorporated with fluorescence. A detection limit of 14 nM was reported.

Dong et al. [53] reported the detection of DNA using a 3-mercaptopropionic acid (MPA)-capped Qdot-MB biosensor probe in which graphene oxide (GO) was used as a fluorescence quencher. Firstly, the Qdot was bonded to the MB and the formed Qdot-MB probe was immobilized on GO. FRET from the Qdot to GO was proposed to be the mode of fluorescence quenching. Their work was the first demonstration of the use of a Qdot-MB probe in which traditional dark fluorescence quenchers were not used, rather a carbon-based nanomaterial was used to modulate the MB operation. The Qdot-based MB was used to detect perfect match, single-base mismatch and three-base mismatch DNA sequences. Their results showed that the detection of the perfect match sequence was 2.5 times higher than the single base mismatch and the fluorescence enhancement response of the three-base mismatch was only 15% that of the perfect complementary DNA target. A limit of detection of 12 nM was reported.

A silica-coated CdSe/ZnS Qdot-MB biosensor was developed by Wu et al. for the detection of DNA [54]. Both perfect complementary and single-base mismatch were detected. Their results showed that the detection of the perfect complementary DNA sequence was ten times more sensitive than the single-base mismatch for low sample DNA concentrations and 2–3 times more sensitive for high DNA concentrations.

β-lactamase gene present in antibiotic-resistance bacteria of plasmids pUC18 in *E. coli* strain DH5α was detected by Wu et al. using a MAA-capped QD-based MB [55]. Two types of MB hybridization strategies using BHQ2 as the quencher were developed, in which the first was a hairpin MB and the second was a double-stranded MB. Direct fluorescence in situ hybridization was used to monitor the hybridization between the target complementary DNA and the probe. The rate of movement of the band and its brightness were used to judge the fluorescence enhancement. It was reported that the hybridization between the target DNA and the Qdot-MB probe was reflected by the increase of the bands brightness. The increase of the fluorescence intensity upon hybridization was reported to be 3.8-fold.

### MicroRNA detection

**Fig. 5:**
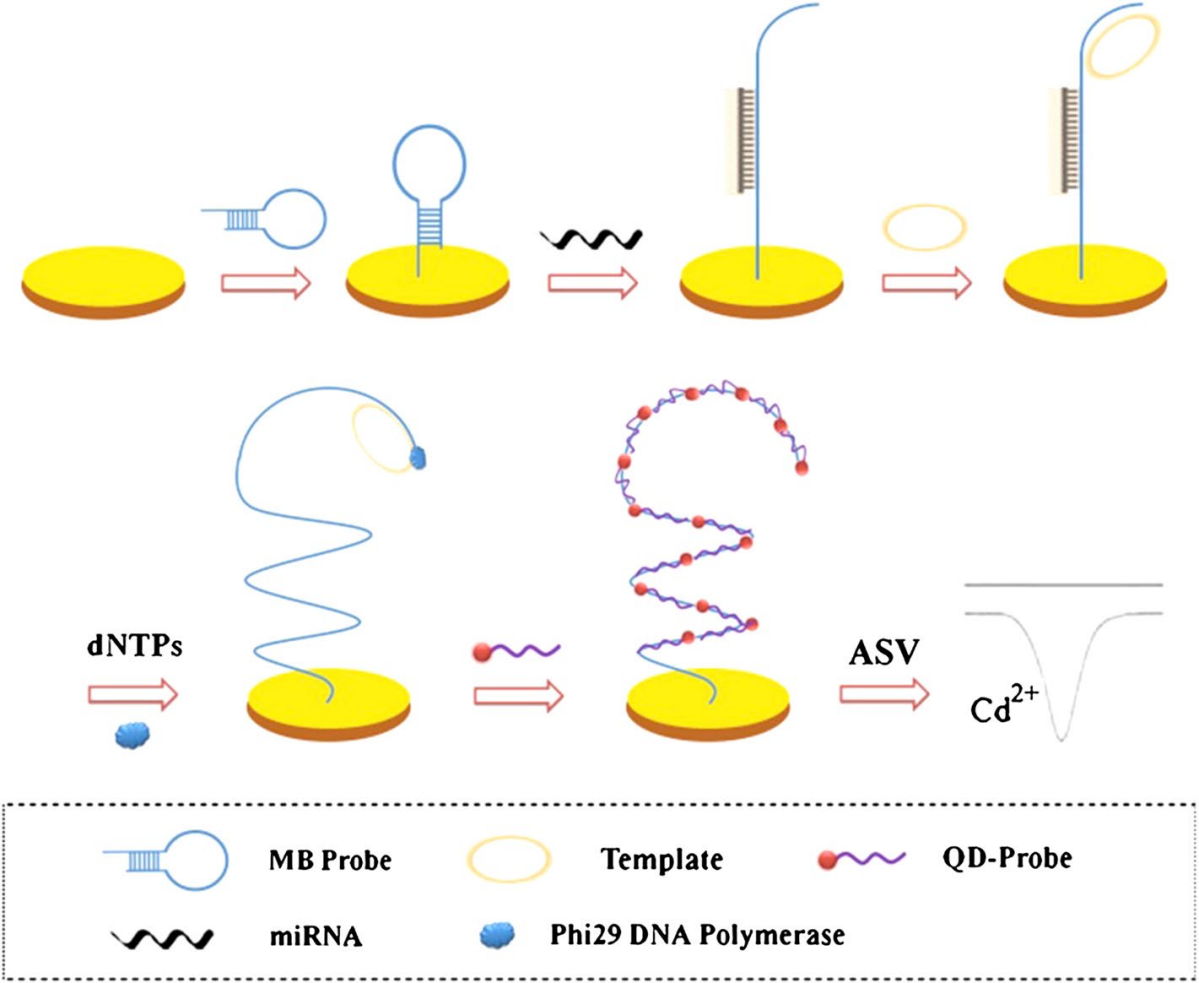
Schematic representation of the designed strategy for miRNA detection. Reprinted from Ref. [56]. Copyright (2013), with permission from Elsevier

An ultrasensitive biosensor for microRNA involving the integration of a MB mediated rolling circle amplification (RCA) and using CdSeTe/CdS Qdot as a fluorophore tag was developed by Wang et al. [56] (Fig. 5). Specifically, miR-16 was selected as the target miRNA. They immobilized a locked nucleic acid (LNA) MB on the surface of a gold electrode and the target miRNA was interacted with the setup to open up the MB. This triggers the LNA-MB loop structure to hybridize with the target miRNA and thus release the RCA primer. Upon binding of the RCA template, Phi29 DNA polymerase and dNTPs were used to initiate the RCA reaction. They obtained a RCA product with thousands of repeated sequences of a long single-stranded DNA which was exploited for linear periodic hybridization when utilizing the Qdot detection probe. They proposed that the Qdot-based RCA product disintegrated into a random DNA coil in aqueous solution as evident from the fluorescence imaging. The low detection limit of 0.32 aM was reported.

Endogenous and exogenous miRNA fluorescence imaging using a Qdot-MB biosensor probe was developed by Lee et al. [57]. The cellular uptake was improved by binding a 9-mer arginine-rich peptide to the Qdots. miRNA124a which is known to be expressed in neurons was chosen as the target. In the absence of the target miRNA124a, the constructed R9-Qdot-miR124a-MB fluorescence was switched OFF while in the presence of the target miRNA124a, the fluorescence was switched ON. Their study is the only reported Qdot-MB biosensor for miRNA imaging.

### Viral RNA detection

RNA-type viruses are an attractive target for Qdot-MB based probes due to the ability to design the loop to target a specific region within the virus RNA genome. It is important to emphasize that detection of viral RNA using MB technology requires the use of highly sensitive Qdot fluorophores. Our research group has recently exploited this technology to detect viral RNA of influenza virus and norovirus. We have reported on the development of a Qdot-MB biosensor probe for influenza virus RNA [46]. In the study, an ultrasensitive thioglycolic acid (TGA)-capped CdZnSeS/ZnSe_1.0_S_1.3_ alloyed Qdot with a fluorescence quantum yield of 98% and exhibiting unique brightness under UV light was synthesized. The synthesis of the Qdot was achieved via engineered band gap alloying. Viral RNA of influenza virus H1N1 in a low concentration range of 2–14 copies/mL was detected both in buffer solution and in human serum. A detection limit of 5.2 copies/mL was reported.

In another related development, an ultrasensitive biosensor for influenza virus H1N1 RNA was developed using a near-infrared quinternary MPA-CdZnSeTeS alloyed Qdot-MB [27]. This biosensor not only detected the viral RNA but it also discriminated between different strains of the virus, (A/Brisbane/59/2007) and (A/California/7/2009). The loop of the MB was complementary to 21 base pairs of (A/Brisbane/59/2007) strain and 22 base pairs of (A/California/7/2009). The Qdot-MB detected the viral RNA both in human serum and in buffer solution. A detection limit as low as ~1 copy/mL was reported.

**Fig. 6:**
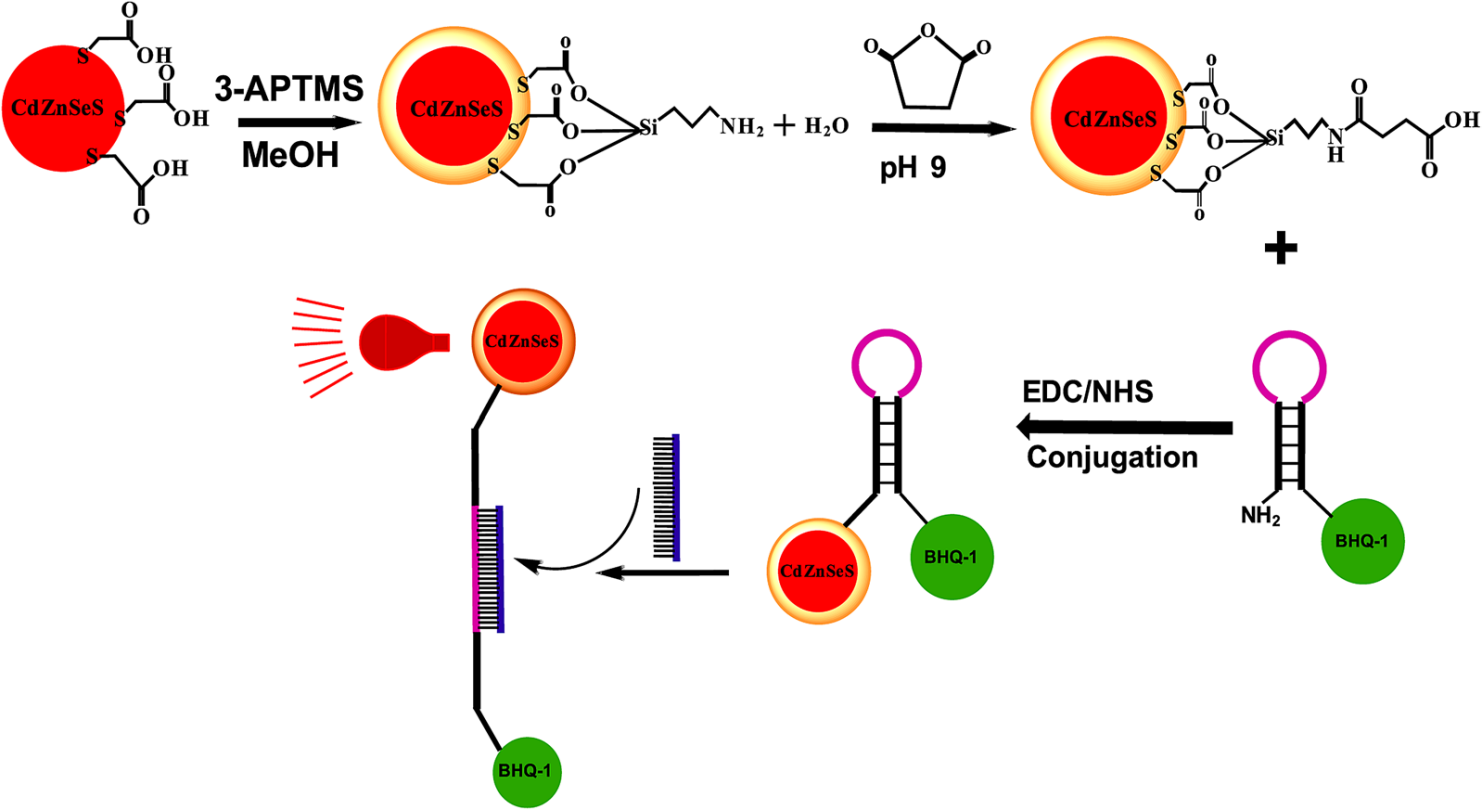
Schematic representation of the ligand-exchange reaction, silica encapsulation and conjugation and detection of NV RNA. The TGA thiol ligand was capped on the surface of the QDs via a ligand-exchange reaction. The QDs were first silanized with an amino group and then further silanized with a carboxylate group. The carboxylate-silanized QDs were conjugated to MB probes via N-(3-dimethylaminopropyl)-N’-ethylcarbodiimide hydrochloride/N-hydroxysuccinimide chemistry. The detection of viral NV RNA was accomplished via hybridization [58]

**Fig. 7:**
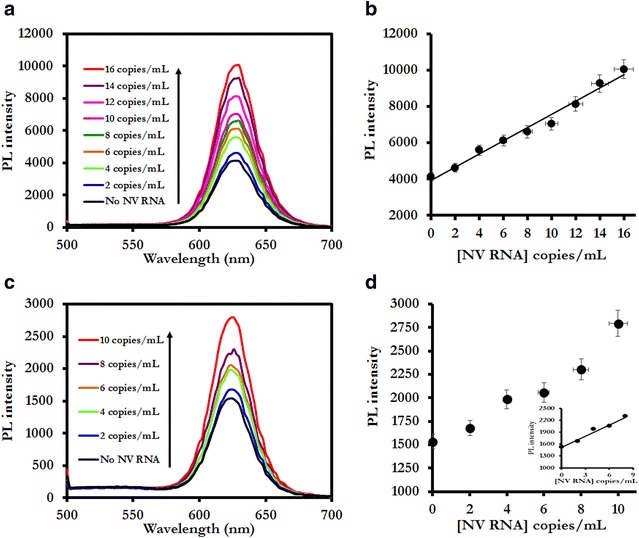
**a** PL-enhancement detection of NV RNA in buffer solution using the SiO_2_-CdZnSeS QD-MB bioprobe and **b** the corresponding PL calibration curve. **c** PL enhancement detection of NV RNA in buffer solution using the TGA-CdZnSeS QD-MB bioprobe and **d** the corresponding PL calibration curve. The *errors bars* represent the standard deviation of three replicate measurements [58]

Norovirus viral RNA detection has been reported using an ultrasensitive silica-functionalized alloyed CdZnSeS Qdot-MB biosensor probe [58]. The synthesis of highly fluorescent SiO_2_-CdZnSeS Qdots as a fluorophore reporter bonded to the MB was achieved via a silanization process in which the surface of TGA-CdZnSeS Qdot was modified (Fig. 6). Norovirus RNA in the concentration range of 2–16 copies/mL was detected in buffer solution and 2–10 copies/mL in human serum (Fig. 7). A detection limit as low as 9.3 copies/mL was reported.

Finally, visualization of viral replication cycle using a self-assembled hexahistidine-anchored Tat peptide Qdot-MB-AuNP biosensor was developed by Yeh et al. [59]. Conventional CdSe/ZnS Qdot was used as the fluorophore reporter and AuNPs was used as the fluorescence quencher. The infectious state of individual host cell was quantitatively monitored both visually and via fluorescence intensity change in the concentration range of 1–200 plaque forming unit.

## Concluding remarks and future outlook

The operational qualities of MB probes with respect to their specific loop design to hybridize with the target nucleic acid sequence and the various optimization flexibilities, suggest that the application of MB within the biological and chemical domain will go on for many years. We cannot ignore the fact that traditional organic fluorescent dyes have been utilized more extensively as fluorophore reporters in MB assays than semiconductor Qdot nanocrystals. However, the optical properties of Qdots are far more superior to those of organic fluorescent dyes. Qdot-based MB probes have been used in DNA, microRNA and viral RNA detection. Even though few Qdot-MB probes have been developed to date, the reported detection limits suggest that unprecedented sensitivity can be achieved. New generation alloyed Qdots with unique optical properties when utilized in MB assay have shown great promise to detect extremely low concentration of RNA viruses. This should open the door for further applications in areas such as pathogen detection, genetic analysis and in protein-nucleic acid interaction. Generally, Cd-based Qdots have been widely utilized due to the ability to tune their optical properties to generate highly fluorescent nanocrystals. Despite this quality, we believe the toxic effect of Cd-based Qdots has discouraged many researchers in exploiting their use in MB assays. Research have shown that the toxicity of Cd-based Qdots is still a debate within the scientific community [60, 61]. It is important to emphasize that there are fabrication strategies available to either eliminate the toxicity or minimize its effect. Shell passivation with non-toxic alloyed semiconductor materials and band gap alloying of Qdots have become mainstream fabrication routes to improve the optical properties of Cd-based Qdots. Qdots produced from these fabrication strategies and which have been applied as fluorescent signal reporters in MB assays have shown great promise based on the unprecedented detection limits that have been reported. Hence, more attention needs to be paid to the use of alloyed Qdots in MB assays. Finally, for those who are still undecided on the use of Cd-based Qdot (with or without non-toxic shell coating or alloying) in MB-based assay, the synthesis of non-cadmium-based fluorescent Qdots with unique optical properties and their use as fluorophore reporter in MB-based assay should be an alternative.
